# Chlorogenic acid improves growth performance of weaned rabbits *via* modulating the intestinal epithelium functions and intestinal microbiota

**DOI:** 10.3389/fmicb.2022.1027101

**Published:** 2022-11-07

**Authors:** Jiali Chen, Zhicheng Song, Rongmei Ji, Yongxu Liu, Hong Zhao, Lei Liu, Fuchang Li

**Affiliations:** ^1^Key Laboratory of Efficient Utilization of Non-grain Feed Resources (Co-construction by Ministry and Province), Ministry of Agriculture and Rural Affairs, Shandong Provincial Key Laboratory of Animal Biotechnology and Disease Control and Prevention, Department of Animal Science, Shandong Agricultural University, Taian, Shandong, China; ^2^Qingdao Kangda Food Co., Ltd., Qingdao, China

**Keywords:** rabbit, chlorogenic acid, growth performance, small intestine, microbiota

## Abstract

This study was conducted to investigate the impacts of chlorogenic acid (CGA) on growth performance, intestinal permeability, intestinal digestion and absorption-related enzyme activities, immune responses, antioxidant capacity and cecum microbial composition in weaned rabbits. One hundred and sixty weaned rabbits were allotted to four treatment groups and fed with a basal diet or a basal diet supplemented with 400, 800, or 1,600 mg/kg CGA, respectively. After a 35-d trial, rabbits on the 800 mg/kg CGA-supplemented group had higher (*p* < 0.05) ADG and lower (*p* < 0.05) F/G than those in control (CON) group. According to the result of growth performance, eight rabbits per group were randomly selected from the CON group and 800 mg/kg CGA group to collect serum, intestinal tissue samples and cecum chyme samples. Results showed that compared with the CON group, supplementation with 800 mg/kg CGA decreased (*p* < 0.05) levels of D-lactate, diamine oxidase, IL-1β, IL-6, and malondialdehyde (MDA), and increased IL-10 concentration in the serum; increased (*p* < 0.05) jejunal ratio of villus height to crypt depth, enhanced (*p* < 0.05) activities of maltase and sucrase, increased (*p* < 0.05) concentrations of IL-10, T-AOC, MHCII and transforming growth factor-α, and decreased (*p* < 0.05) levels of TNF-α and MDA in the jejunum of weaned rabbits. In addition, results of high-throughput sequencing showed that CGA supplementation elevated (*p* < 0.05) microbial diversity and richness, and increased (*p* < 0.05) the abundances of butyrate-producing bacteria (including genera *V9D2013_group*, *Monoglobus*, *Papillibacter*, *UCG-005*, and *Ruminococcus*). These results indicated that dietary supplementation with 800 mg/kg CGA could improve the growth performance of weaned rabbits by enhancing intestinal structural integrity, improving the intestinal epithelium functions, and modulating the composition and diversity of gut microbiota.

## Introduction

Weaning is an inevitable and stressful process for mammals, as it may affect the morphological structure, barrier function, and intestinal microbiota of the gastrointestinal tract with altered food status, resulting in reduced feed intake and growth retardation, and consequent economic losses (Gharib et al., 2018). Moreover, accumulated evidences showed that weaning stress could cause mammalian intestinal barrier dysfunction by inducing physiological inflammation and oxidative stress within a short time after weaning (Smith et al., 2010; Hu et al., 2013; Gharib et al., 2018). It is reported that digestive diseases account for 70% of all rabbit diseases (Carabaño et al., 2008) and up to 60% mortality were cause by epizootic rabbit enteropathy, especially for weaned rabbits (Fann and O'Rourke, 2001; Carabaño et al., 2008). As we all know, intestine is not only the main site for nutrient absorption but also provides a primary barrier against pathogenic microorganisms (Martens et al., 2018). Therefore, it is a priority to explore effectively and safely strategies to prevent gastrointestinal mucosal damage in weaned rabbits.

Plant polyphenols have been reported to be able to ameliorate the damage of animals induced by stress (Relja et al., 2012; Yan et al., 2020). Chlorogenic acid (CGA) is one kind of natural polyphenols that formed by esterification of caffeic acid and quinic acid and widely found in coffee, fruits, and vegetables (Bouayed et al., 2007). It is safe with minimal side effects (Metwally et al., 2020) and possesses various biological properties, including antioxidant, anti-inflammatory, and antimicrobial activities, all of which contributed to maintain intestinal health (Chen et al., 2018b, 2021b). Previous study showed that CGA could restore intestinal epithelial tight-junction integrity and ameliorate intestinal inflammation in lipopolysaccharide (LPS)-challenged Caco-2 cells (Yu et al., 2021) and enhance the growth performance of weaned rats (Ruan et al., 2014). Moreover, an early study in weaned pigs showed that dietary CGA supplementation could improve the growth performance by enhancing the intestinal digestion and absorption function (Chen et al., 2018a). However, until now, there is little literature available evaluating the growth-promoting effect of CGA on weaned rabbits whose intestine is more vulnerable and sensitive to weaning stress. In addition, as CGA is partially consumed by symbiotic microorganisms in the large intestine to generate short-chain fatty acids (Lu et al., 2020), it has the potential to be employed as prebiotics to repair damaged intestinal barrier (Chen et al., 2019).

Therefore, in view of the foregoing, it is plausible to hypothesize that CGA has the potential in enhancing the growth performance of weaned rabbits by maintaining the intestinal barrier structure and function. This study was carried out to verify the above hypothesis by evaluating the effects of CGA on intestinal permeability, intestinal digestion and absorption-related enzyme activities, immune responses, antioxidant capacity and cecum microbial composition in weaned rabbits.

## Materials and methods

### Experimental animals, diet and management

A total of one hundred and sixty weaned rabbits, with an initial average body weight (BW) of 1.05 ± 0.01 kg, were randomly allotted to four dietary treatments (*n* = 40) in a randomized complete block design. The treatments consist of a basal diet (control, CON) and a basal diet supplemented with 400, 800, or 1,600 mg/kg CGA (provided by Chengdu Hengfeng Tiancheng Technology Co., Ltd., Chengdu, China). The experiment lasted for 35 days. The basal diet was formulated based on the recommendation of Mateos et al. (2010), and the feed was made into 4-mm pellets by using steam. Ingredients and compositions of the basal diet are shown in Table 1. All rabbits were housed in cages (60 cm× 40 cm × 40 cm) individually and were given *ad libitum* access to fresh water and feed. Temperature and lighting were maintained according to commercial conditions (20 to 23°C, 12 light/12 dark).

**Table 1:** Composition and nutrient levels of basal diets (air-dry basis).

Ingredients	Content (%)	Nutrient levels^2^	Content (%)
Corn	13.30	Dry matter	84.49
Bean pulp	13.00	Crude protein	16.38
Wheat bran	19.00	Crude fat	3.10
Corn germ meal	19.00	Crude fiber	15.44
Alfalfa meal	12.00	Acid detergent fiber	20.60
Soya bean stem meal	19.00	Neutral detergent fiber	37.85
Soya oil	0.70	Ash	5.36
Premix^1^	4.00	Calcium	1.13
Total	100.00	Phosphorus	0.49

^1^The following premix ingredients were added to each kg of diet: vitamin A, 8,000 IU; vitamin D3, 1,000 IU; vitamin E, 50 mg; vitamin K3, 2.3 mg; thiamine, 1.75 mg; riboflavin, 6.9 mg; niacin, 28.45 mg; pantothenic acid, 6.7 mg; biotin, 2.75 mg; folic acid, 0.6 mg; vitamin B12, 2.2 mg; choline, 420 mg; lysine, 1.5 g; methionine, 1.5 g; copper, 50 mg; iron, 100 mg; manganese, 30 mg; magnesium, 150 mg; iodine, 0.1 mg. ^2^Values were calculated.

### Growth performance

Feed consumption of each rabbit were measured daily throughout the trial. The BWs of rabbits were individually measured after 12 h fasting on the morning of days 1, 15 and 36 of the feeding trial. The value of average daily BW gain (ADG), average daily feed intake (ADFI), and the feed-to-gain ratio (F/G) were then calculated.

### Sampling collection and preparation

According to the result of growth performance, 800 mg/kg feed is the optimum dose of CGA for growth promotion. Therefore, to further explain the growth-promoting effect of CGA, eight rabbits with the average BW of CON and 800 mg/kg CGA treatment groups were selected for sample collection. Blood samples were collected from the ear edge vein, and serum samples were then harvested after centrifugation at 3,000× *g* for 10 min at 4°C and stored at-20°C for further analysis. The same sixteen rabbits were then sacrificed by cervical dislocation, and the abdomen was immediately opened to remove the entire small intestine. Two segments (about 2-cm) of the mid-jejunum were quickly isolated, and washed gently by cold saline solution. One segment was fixed in 4% paraformaldehyde for morphology measurements; another segment was divided into different packages, and stored at-80°C for further analysis after being rapidly frozen in liquid nitrogen. Furthermore, the chyme samples of cecum were quickly collected as described in Li et al. (2019), and stored at-80°C for microbiological analysis.

### Intestinal morphology measurement

The jejunum morphology was conducted as previously described by Li J. et al. (2022). After fixation for 24 h, jejunal segments were removed from the 4% paraformaldehyde solution, dehydrated with normal saline, and embedded in paraffin wax. Tissue sections were cut into ~5 μm slices using a microtome (Leica RM2235), followed by being fixed on slides, and stained with hematoxylin and eosin. Jejunum mucosal morphology including villus height and crypt depth was determined with ImageJ analysis software (Version 1.47, Bethesda, MD, United States). A minimum of 10 well-orientated villi and their adjoined crypts from each sample were chosen for statistical analysis.

### Serum cytokines, antioxidant parameters, diamine oxidase (DAO) and D-lactic acid levels

The serum levels of serum interleukin 1 beta (IL-1β), tumor necrosis factor-alpha (TNF-α), interleukin 10 (IL-10), interleukin 6 (IL-6) and D-lactic acid were measured spectrophotometrically by the corresponding rabbit enzyme-linked immunosorbent assay (ELISA) kit (Jiangsu Meimian Industrial Co., Ltd., Jiangsu, China) according to the previous study (Li J. et al., 2022). The concentrations of malondialdehyde (MDA), total antioxidant capacity (T-AOC), glutathione peroxidase (GSH-Px), catalase (CAT) and diamine oxidase (DAO) in serum were measured by corresponding assay kits (Nanjing Jiancheng Institute of Bioengineering, Nanjing, China) according to the manufacturer’s instructions.

### Jejunum mucosa parameters measurements

The mucosa samples of jejunum were homogenized with ice-cold saline solution in a weight (g): volume (ml) ratio of 1: 9. The homogenates were centrifuged at 2,500 × g for 10 min at 4°C, and the jejunum mucosal supernatant was collected for the further detection. The concentration of intestinal mucosa cytokines (IL-1β, TNF-α, IL-10 and IL-6), transforming growth factor-α (TGF-α), trefoil factor family (TFF) and major histocompatibility complex II (MHCII) were measured by the corresponding rabbit ELISA kit (Jiangsu Meimian Industrial Co., Ltd., Jiangsu, China) according to the previous study (Li J. et al., 2022). The concentration of disaccharide enzyme (lactase, maltase and sucrase) and antioxidant indexes (MDA, T-AOC, GSH-Px and CAT) were measured by the corresponding detection kits purchased from Nanjing Jiancheng Institute of Bioengineering (Nanjing, China) according to the manufacturer’s instructions.

### 16S rRNA sequencing and analysis

Microbial composition and diversity were analyzed as described in Li et al. (2020). Briefly, microbial DNA was extracted from frozen fecal samples using an E.Z.N.A.™ Stool DNA kit (Omega Bio-Tek, Norcross, GA, United States) according to the manufacturer’s protocols. After the final DNA concentration and purity determining, DNA quality was checked by 1% agarose gel electrophoresis. DNA was diluted to 1 ng/μL using sterile water, and the V4 hypervariable regions of the bacterial 16S rRNA gene were amplified with the primers 515F (5′-GTGCCAGCMGCCGCGGTAA-3′) and 806R (5′-GGACTACHVGGGTWTCTAAT-3′) on the Illumina HiSeq PE2500 platform by Novogene (Beijing, China). The filtered, non-chimeric high-quality sequences (tags) were clustered into the same operational taxonomic units (OTUs) by 97% sequence similarity using UPARSE software. Then the OTUs were classified to different taxonomic levels using SILVA database based on Mothur algorithm to annotate taxonomic information. Alpha diversity and beta diversity were analyzed after the operational taxonomic unit abundance information were normalized. Shannon, Simpson, Chao 1, and ACE indexes were chosen to ascertain differences in alpha diversity between the different groups (Chen L. et al., 2018; Gallego et al., 2021), and Bray-Curtis distances were calculated for comparison of taxonomic data in beta diversity, and visualized using Principal Coordinate Analysis (PCoA; Lozupone and Knight, 2005). The statistical differences in alpha and beta diversity of bacterial communities between the two groups were examined using the Wilcoxon rank-sum test. The analysis of similarity (ANOSIM) test method was used to access the significant difference among the microbial communities.

### Statistical analysis

All data were assessed for normal distribution using the Shapiro–Wilk’s statistic (W > 0.05). Data on growth performance were analyzed using one-way analysis of variance (ANOVA) procedure of SAS 9.4 (Institute Inc., Cary, NC), followed by Tukey’s multiple-range test to compare the statistical differences among groups. The linear and quadratic responses to the dietary CGA levels were detected by using the REG procedure in SAS. The results of the experiments were expressed as mean and SEM. Statistical significance was presented at *p* < 0.05 and a tendency toward difference was considered as *p* < 0.10. For other indexes, data were analyzed by *T*-test using the statistical program SAS 9.4. Spearman’s rank test was used to evaluate the correlations between the abundances of differential bacteria and the levels of inflammatory cytokines and antioxidant indices. Multiple testing was corrected by using the Benjamini-Hochberg false discovery rate. The data were plotted in the figures as mean and standard error. Significant differences are displayed in the figures by ^*^0.01 < *p* < 0.05, and ^**^p < 0.01, while 0.05 < *p* < 0.10 was considered as a trend to significance.

## Results

### Growth performance

As shown in Table 2, higher (*p* < 0.05) BW on day 35 of the trial was observed in rabbits fed the 800 mg/kg CGA-supplemented diet compared with those fed the CON and 1,600 mg/kg CGA-supplemented diets and demonstrated quadratic (*p* < 0.05) effects in response to increasing CGA levels. However, dietary CGA supplementation had no significant effect (*p* > 0.05) on the growth performance of weaned rabbits on days 1–14 of the trial. From days 15 to 35, the rabbits fed CGA-supplemented diets tended (*p* < 0.10) to have an increased ADFI compared with those fed the CON diet; the ADG in 400 and 800 mg/kg CGA-supplemented groups was higher (*p* < 0.05) than that in CON group; The ADG in 800 mg/kg CGA-supplemented group was higher (*p* < 0.05) than that in 1600 mg/kg CGA-supplemented group and showed a quadratic (*p* < 0.05) effect in response to increasing CGA levels; but no significant difference (*p* > 0.05) was observed in F/G among the groups. For the whole trial period, the ADG of rabbits in 800 mg/kg CGA-supplemented group were higher (*p* < 0.05) than that in CON and 1,600 mg/kg CGA-supplemented groups; lower (*p* < 0.05) F/G was observed in rabbits fed the 800 mg/kg CGA-supplemented diet compared with those fed the CON and 1,600 mg/kg CGA-supplemented diets; the ADG and F/G demonstrated quadratic (*p* < 0.05) effects in response to increasing CGA levels; in addition, dietary supplemented with CGA tended (*p* < 0.10) to affect the ADFI of rabbits.

**Table 2:** Effects of CGA on growth performance of weaned rabbits.

	Dietary CGA levels (mg/kg)					*p*-Values		
Items	0	400	800	1,600	SEM	Treatment	Linear	Quadratic
Initial BW (kg)	1.05	1.04	1.05	1.05	0.012	0.996	0.998	0.985
d 14 BW (kg)	1.58	1.58	1.64	1.55	0.018	0.308	0.694	0.121
d 35 BW (kg)	2.47^b^	2.59^ab^	2.66^a^	2.46^b^	0.027	0.018	0.734	0.002
d 1–14								
ADFI (g/d)	124.59	125.83	128.59	121.42	1.635	0.486	0.495	0.189
ADG (g/d)	38.39	38.37	44.03	36.23	1.201	0.115	0.670	0.057
F/G	3.47	3.68	3.11	3.78	0.103	0.101	0.494	0.148
d 15–35								
ADFI (g/d)	155.38	166.91	166.54	156.25	2.154	0.090	0.813	0.014
ADG (g/d)	42.43^c^	47.99^ab^	48.34^a^	43.18^bc^	0.886	0.023	0.911	0.002
F/G	3.78	3.52	3.55	3.76	0.058	0.243	0.862	0.050
d 1–35								
ADFI (g/d)	143.07	150.48	151.36	142.32	1.553	0.067	0.628	0.009
ADG (g/d)	40.81^b^	44.14^ab^	46.61^a^	40.40^b^	0.775	0.010	0.733	0.001
F/G	3.61^a^	3.45^ab^	3.31^b^	3.63^a^	0.046	0.048	0.843	0.008

Results are presented as mean and SEM. ^ab^Means in the same row with different letter differ (*P* < 0.05). *n* = 40. BW, bodyweight; ADFI, average daily feed intake; ADG, average daily gain; F/G, feed efficiency (the ratio of ADFI and ADG).

### Intestinal morphology

**Figure 1:**
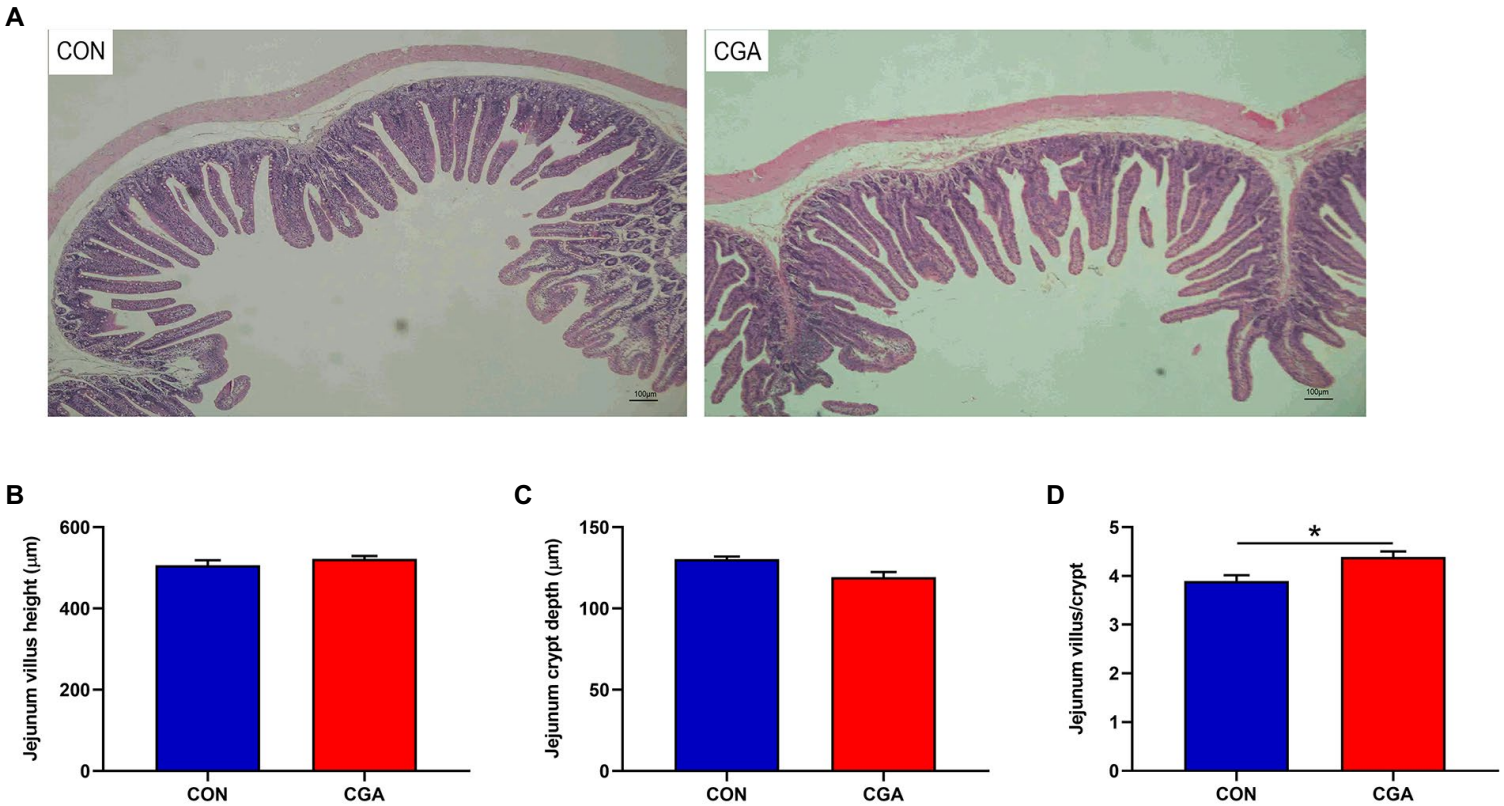
Effects of CGA on intestinal morphology in the jejunum of weaned rabbits. **(A)** Hematoxylin and eosin photomicrographs obtained at 100 × magnification; **(B)** Villus height; **(C)** Crypt depth; **(D)** Villus height/crypt depth ratio. CON, rabbits fed a basal diet; CGA, rabbits fed the basal diet supplemented with 800 mg/kg chlorogenic acid. Results are presented as mean and SEM (*n* = 8). Significant difference was recorded by 0.01 < *p* < 0.05*.

The intestinal morphology of rabbits is showed in Figure 1. Rabbits in CGA group had higher (*p* < 0.05) villus height to crypt depth ratio than rabbits in CON group. However, no significant effect of dietary CGA supplementation was detected on villus height and crypt depth.

### Intestinal barrier and function

**Figure 2:**
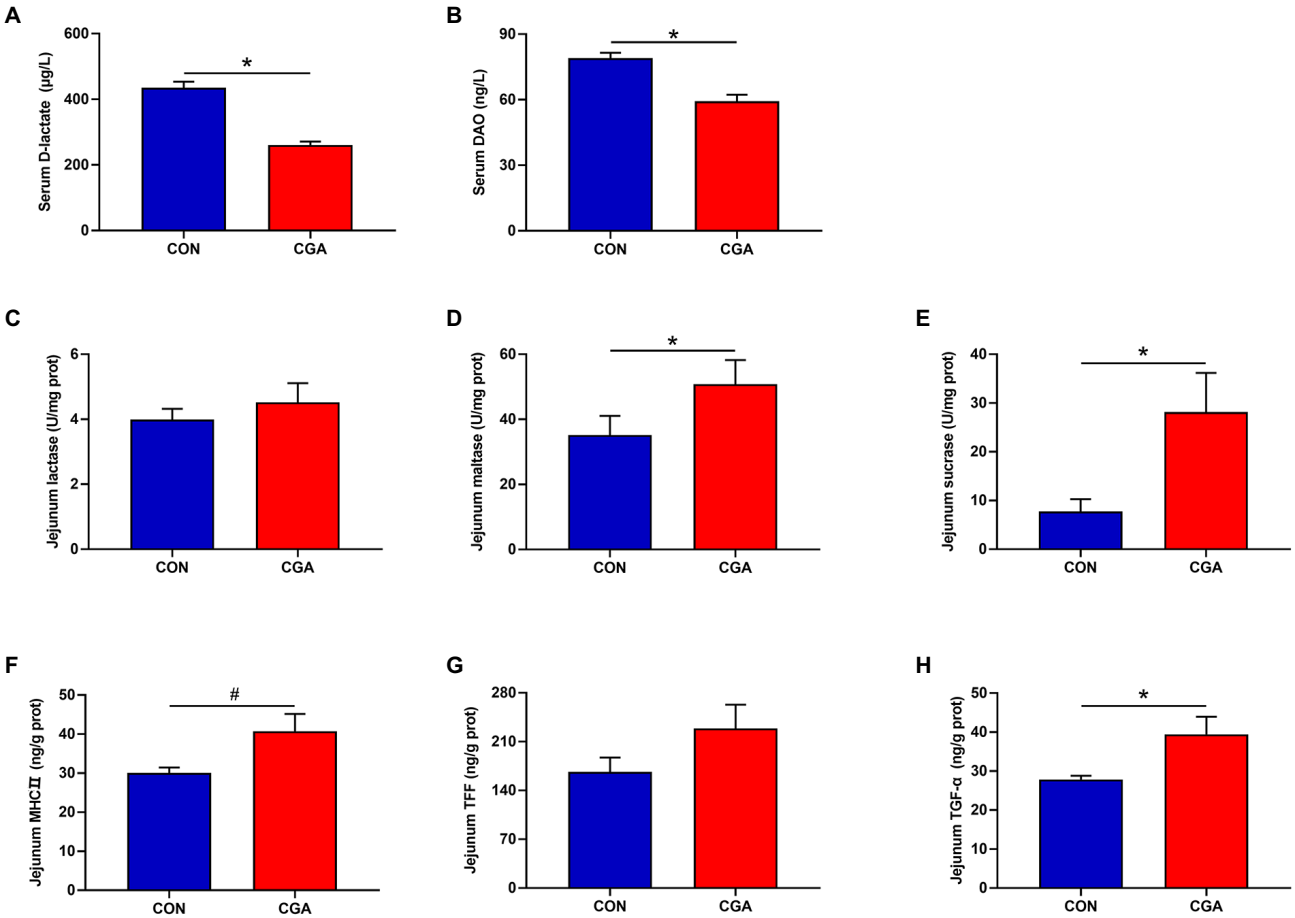
Effects of CGA on the maturity and integrity of small intestine in weaned rabbits. **(A)** serum D-lactate; **(B)** serum diamine oxidase (DAO); **(C)** jejunal lactase; **(D)** jejunal maltase; **(E)** jejunal sucrase; **(F)** jejunal major histocompatibility complex II (MHCII); **(G)** jejunal trefoil factor family (TFF); **(H)** jejunal transforming growth factor-α (TGF-α). CON, rabbits fed a basal diet; CGA, rabbits fed the basal diet supplemented with 800 mg/kg chlorogenic acid. Results are presented as mean and SEM (*n* = 8). Significant difference was recorded by 0.01 < *p* < 0.05*. ^#^Means a tendency to difference (0.05 < *p* < 0.10).

As shown in Figure 2, the levels of DAO (Figure 2A) and D-lactase (Figure 2B) in the serum were decreased (*p* < 0.05) by CGA supplementation. Compared with the CON group, CGA supplementation increased (*p* < 0.05) the activities of maltase (Figure 2D) and sucrase (Figure 2E) in the jejunum mucosa of rabbits. However, there was no significant difference in lactase (Figure 2C) activity between the two groups. Besides, higher (*p* < 0.05) TGF-α (Figure 2H) concentration in the jejunum mucosa was also observed in rabbits fed the CGA-supplemented diet, meanwhile rabbits fed CGA diet tended to (0.05 < *p* < 0.10) have an increased jejunum MHCII (Figure 2F) concentration. But no significant difference was observed in jejunal concentration of TFF (Figure 2G) in rabbits between the two groups.

### Systemic and intestinal inflammatory cytokines

**Figure 3:**
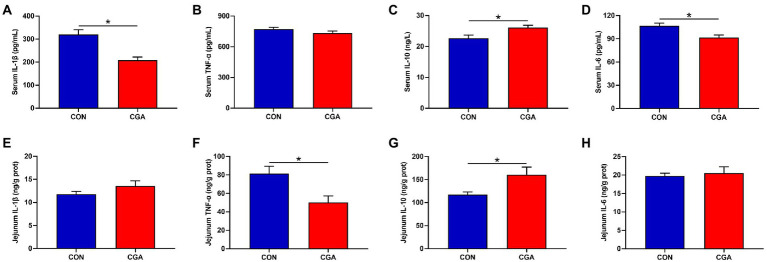
Effects of CGA on the levels of critical inflammation-related molecules in the serum **(A–D)** and jejunum **(E–H)** of weaned rabbits. IL-1β, interleukin 1 beta; TNF-α, tumor necrosis factor-alpha; IL-10, interleukin 10; IL-6, interleukin 6; CON, rabbits fed a basal diet; CGA, rabbits fed the basal diet supplemented with 800 mg/kg chlorogenic acid. Results are presented as mean and SEM (*n* = 8). Significant difference was recorded by 0.01 < *p* < 0.05*.

The effects of CGA on serum and jejunum inflammatory cytokines are presented in Figure 3. Compared with the CON group, the serum IL-1β (Figure 3A) and IL-6 (Figure 3D) levels were decreased (*p* < 0.05), whereas the serum IL-10 (Figure 3C) level was increased (*p* < 0.05) in CGA group. Meanwhile, rabbits fed the CGA diet had lower (*p* < 0.05) TNF-α (Figure 3F) level and higher (*p* < 0.05) IL-10 (Figure 3G) level in the jejunum were observed in rabbits on CGA group. However, no significant differences in serum TNF-α (Figure 3B) concentration and jejunal IL-1β (Figure 3E) and IL-6 (Figure 3H) levels were detected between the two treatment groups.

### Systemic and intestinal antioxidant capacity

**Figure 4:**
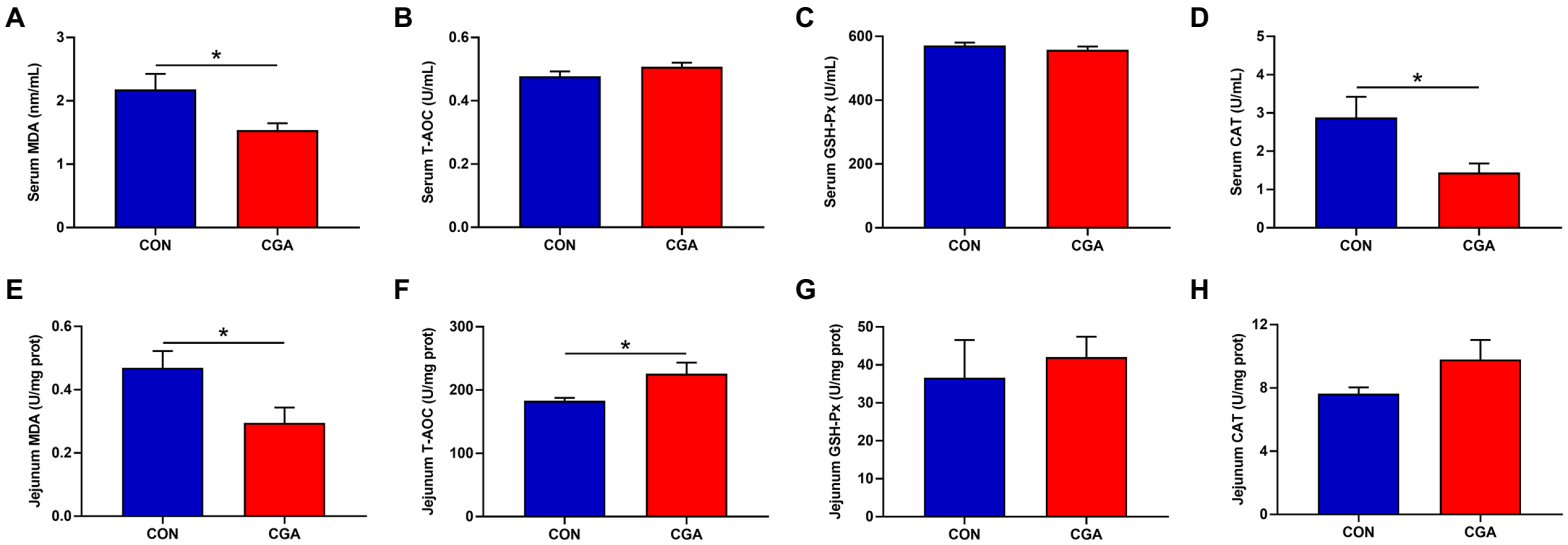
Effects of CGA on the levels of antioxidant indicators in the serum **(A–D)** and jejunum **(E–H)** of weaned rabbits. MDA, malondialdehyde; T-AOC, total antioxidant capacity; GSH-Px, glutathione peroxidase; CAT, catalase; CON, rabbits fed a basal diet; CGA, rabbits fed the basal diet supplemented with 800 mg/kg chlorogenic acid. Results are presented as mean and SEM (*n* = 8). Significant difference was recorded by 0.01 < *p* < 0.05*.

As showed in Figure 4, dietary CGA supplementation decreased (*p* < 0.05) the activity of CAT (Figure 4D) and the content of MDA (Figure 4A) but had no significant effects on the activities of T-AOC (Figure 4B) and GSH-Px (Figure 4C) in the serum of rabbits. In jejunum, the content of T-AOC (Figure 4F) was increased (*p* < 0.05) and the MDA (Figure 4E) content was decreased (*p* < 0.05) by CGA supplementation. There were no significant differences in the activities of GSH-Px (Figure 4G) and CAT (Figure 4H) between the two treatment groups.

### Analysis of bacterial community and diversity

**Figure 5:**
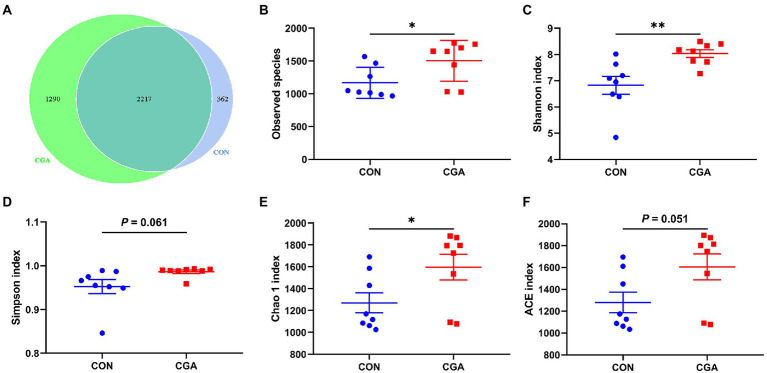
Differences on bacteria community diversity and richness between the two groups. **(A)** A Venn diagram generated to depict shared and nique sequences between the two groups; **(B)** Observed species; **(C)** Shannon index; **(D)** Simpson index; **(E)** Chao 1 index; **(F)** ACE index. CON, rabbits fed a basal diet; CGA, rabbits fed the basal diet supplemented with 800 mg/kg chlorogenic acid. Results are presented as mean and SEM (*n* = 8). Significant difference was recorded by 0.01 < *p* < 0.05*, *p* < 0.01**.

As shown in Supplementary Figure 1, a total of 1,140,800 total tags, 1,067,486 taxon tags, 74 unclassified tags, and 73,240 unique tags were obtained from 16 cecal digesta samples. After taxonomic information was annotated, a total of 23,085 OTUs were obtained, with an average of 1,272 ± 92 in CON group and 1,614 ± 118 in CGA group. The bacteria community diversity and richness are shown in Figure 5. The two groups shared 2,217 common OTUs, and rabbits in CGA group showed an increased number of unique OTUs (Figure 5A). Compared with the CON group, CGA group showed significantly higher (*p* < 0.05) observed species (Figure 5B), Shannon index (Figure 5C), and Chao 1 index (Figure 5E), and tended to (0.05 < *p* < 0.10) increase Simpson index (Figure 5D) and ACE index (Figure 5F) of cecal microbiota in weaned rabbits.

**Figure 6:**
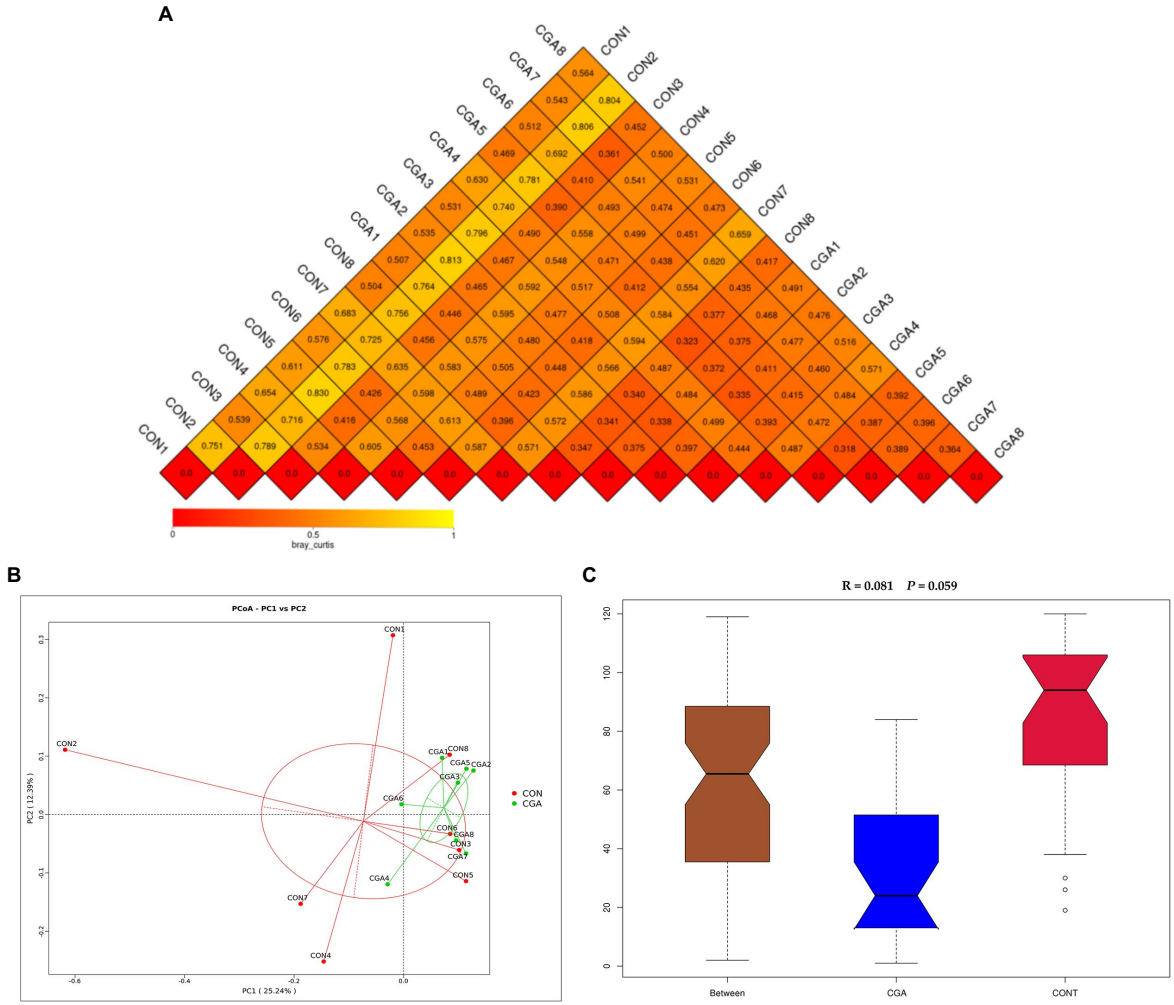
Beta diversity of microbial community analysis. **(A)** Heat-map of beta diversity for each two samples in the two groups by weighted Unifrac distance. **(B)** The principal coordinate analysis (PCoA) profile of weighted Unifrac distance. **(C)** Analysis of ANOSIM. *R* value is scaled to lie between −1 and + 1. Generally, 0 < *R* < 1 and *p* < 0.05 represents that there were significant differences between the groups. CON, rabbits fed a basal diet; CGA, rabbits fed the basal diet supplemented with 800 mg/kg chlorogenic acid. *n* = 8.

The beta diversity of microbial community is shown in Figure 6. The results of heat-map (Figure 6A) and PCoA plot (Figure 6B) drawn based on the bray_curtis distances displayed that the PFA samples tended to cluster separately from the CON samples, and ANOSIM (Figure 6C) showed the two groups tended to have different bacterial community structures (R = 0.081, *p* = 0.059).

### Relative abundance of cecal microbiota

**Figure 7:**
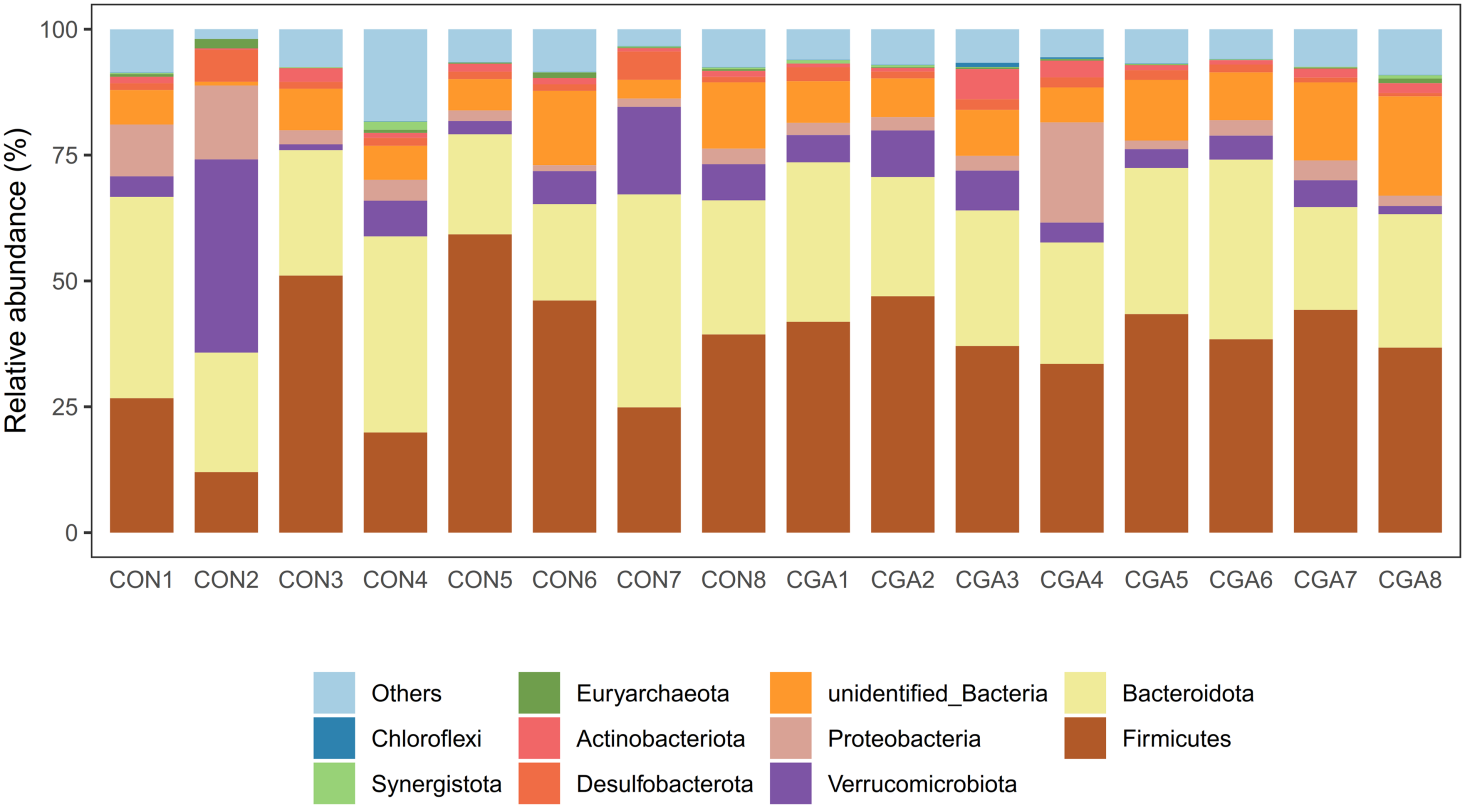
Bar graph shows the phylum level composition of bacteria. Color-coded bar plot shows the relative abundance of bacterial phylum across the different samples. CON 1, 2, 3, 4, 5, 6, 7, 8 are cecal digesta samples from rabbits fed with a basal diet; CGA 1, 2, 3, 4, 5, 6, 7, 8 are cecal digesta samples from rabbits fed with a basal diet supplemented with 800 mg/kg chlorogenic acid.

The top 10 phyla in relative abundance of cecal microbiota are shown in shown in Figure 7 and Supplementary Table S1. The most predominant phyla in cecal samples of rabbits were Firmicutes and Bacteroidetes. However, no significant differences were observed in the relative abundances of top ten phyla between the two groups.

**Figure 8:**
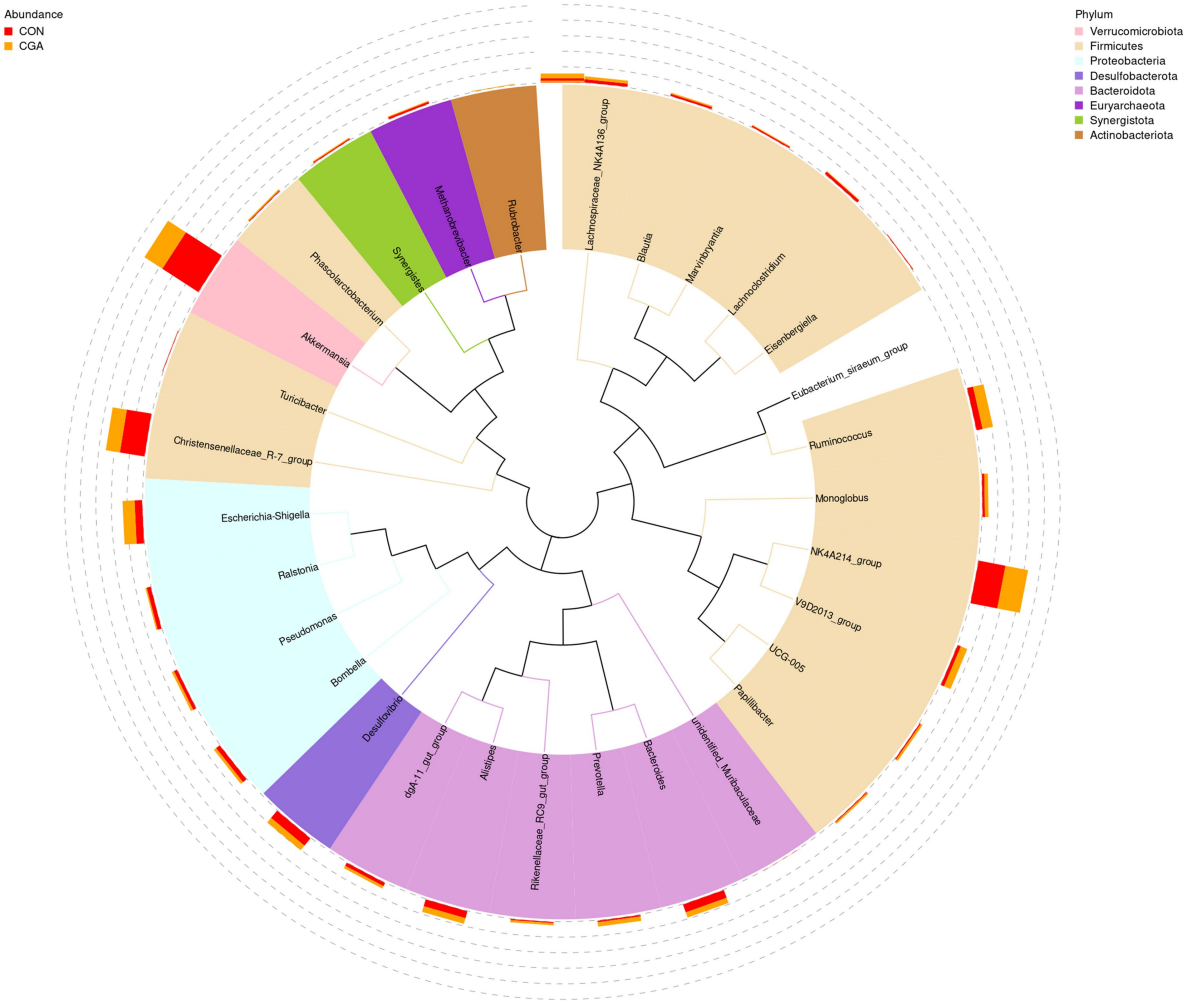
Changes of the relative abundance at genus levels. The phylogenetic tree constructed based on the sequence of the top 35 genera. The branches with different colors in the inner circle represent their corresponding phylum, and the stacked column chart in the outer circle indicates the relative abundance of each genus in different treatments. CON, rabbits fed a basal diet; CGA, rabbits fed the basal diet supplemented with 800 mg/kg chlorogenic acid.

The relative abundances at genus level in rabbit cecal microbiota (top 30 genera) are shown in Figure 8 and Supplementary Table S2. The identified most plentiful genera in cecal samples were *Akkermansia*, *NK4A214_group*, and *Christensenellaceae_R-7_group*. Of the top 35 genera, the relative abundances of *V9D2013_group*, *Monoglobus*, *Papillibacter*, and *UCG-005* were higher (*p* < 0.05) in CGA group than in CON group. Besides, dietary CGA supplementation tended to increase the relative abundance of *Ruminococcus* compared with the CON group (*p* = 0.095).

### Correlation analysis between the abundances of differential bacteria and the levels of inflammatory cytokines and antioxidant indices

**Figure 9:**
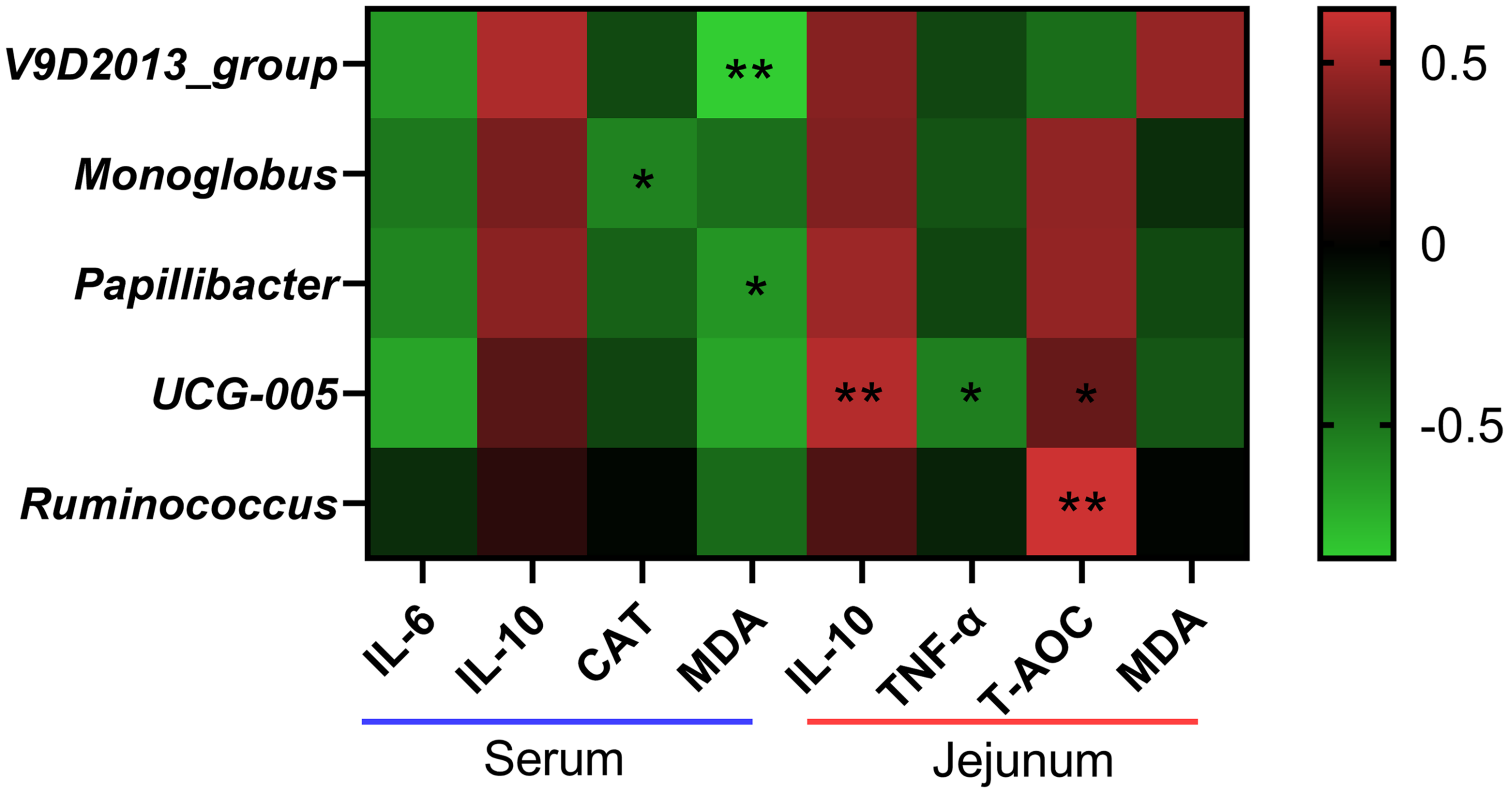
Correlation analysis between the abundances of differential bacteria at genus level and the levels of inflammatory cytokines and antioxidant indices. IL-6, interleukin 6; IL-10, interleukin 10; CAT, catalase; MDA, malondialdehyde; TNF-α, tumor necrosis factor-alpha; T-AOC, total antioxidant capacity. *the correlation is significant at a level of 0.05. **the correlation is significant at a level of 0.01.

As shown in Figure 9, the abundances of *V9D2013* and *Papillibacter* showed negative correlation with the serum MDA concentration (*p* < 0.05). The abundance of *Monoglobus* had significant positive correlation with the CAT activity (*p* < 0.05). The abundance of *UCG-005* showed positive correlations with jejunal IL-10 and T-AOC levels (*p* < 0.05), and negative correlations with jejunal TNF-α concentration (*p* < 0.05). In addition, the abundance of *Ruminococcus* had significant positive correlation with jejunal T-AOC concentration (*p* < 0.05).

## Discussion

Numerous studies have verified the beneficial effects of CGA on the health of human beings and animals (Naveed et al., 2018; Lu et al., 2020). The present study showed that dietary supplemented with 800 mg/kg CGA increased the ADG and F/G in rabbits though had no significant effect on feed intake, which was in accordance with previous studies in weaned pigs and rats (Ruan et al., 2014; Chen et al., 2018a). It suggested that dietary supplementation with 800 mg/kg CGA could increase the bioavailability of dietary nutrients and CGA can also play a good growth promoter for weaned rabbits. These interesting results provided a foundation for further study and the application of CGA in rabbits.

The small intestine is the leading site for nutrient digestion and absorption. A previous study has indicated that the complete intestinal structure is the basis for its normal function (Duan et al., 2022). Maintenance of normal small intestine morphology and structural integrity are imperative for improving nutrient absorption and preventing bacterial translocation from the intestinal tract. However, weaning stress often induces dramatic changes in intestinal morphology, such as villous shedding, villus atrophy, and crypt hyperplasia (Bivolarski and Vachkova, 2014), which adversely influences the digestion and absorption of nutrients and consequently causes growth retardation (Cera et al., 1988). The villi are critical components of the digestive tract, and determine the absorption capacity of the small intestine (Heo et al., 2013). Moreover, the ratio of villus height to crypt depth is an important parameter in evaluating the absorptive capacity of the small intestine (Xu et al., 2017). The findings of Ruan et al. (2014) showed that CGA significantly increased the villus height and the ratio of villus height to crypt depth, and decreased the crypt depth in the jejunum of LPS-challenged weaned rodents. Similarly, in present study, we found that the small intestinal mucosal morphology was significantly elevated by increasing the ratio of villus height to crypt depth in the jejunum, suggesting that dietary CGA supplementation could maintain favorable mucosa structure and enlarge the absorptive area of luminal villous, which benefited to promote the intestinal health of weaned rabbits.

Changes of the intestinal morphology is usually correlated to decreased digestion and absorption function, mainly characterized by digestive enzyme alteration (Kirmiz et al., 2018). Disaccharide enzymes, including maltase, lactase and sucrose, are the main enzymes in brush border of the intestinal epithelium, which play important roles in promoting carbohydrate breakdown, providing continuous energy reserves, and then promoting the growth performance (Fleige et al., 2007). In this study, 800 mg/kg CGA supplementation significantly improved the activities of maltase and sucrase in the jejunum of weaned rabbits, implying that dietary supplementation with 800 mg/kg CGA helped to improve digestion function of small intestine in weaned rabbits. Similarly, Chen et al. (2018a) also found that dietary supplemented 1,000 mg/kg CGA enhanced jejunal activities of lactase and maltase in weaned piglets. Moreover, previous studies have indicated that phenolic compounds (including CGA) possess potential roles in regulating nutrient (such as protein and lipid) metabolism in liver tissues (Lafay et al., 2006; Bagdas et al., 2020). These results in current study, together with similar results, might be a possible explanation for the positive effects of CGA on the growth performance of CGA-supplemented weaned rabbits.

The integrity of intestinal barrier is closely related to the maintenance of intestinal function. However, previous studies in mammal indicated that weaning stress could induce seriously impairment of small intestinal barrier function within a short time after weaning (Smith et al., 2010; Bivolarski and Vachkova, 2014). It is well known that intestinal permeability can be commonly assessed using some blood indices. Diamine oxidase, an intracellular enzyme, is primarily expressed in the small intestine, but rare in the serum under normal circumstances (Zhao et al., 2014). Once the intestinal epithelial barrier is injured, the serum DAO levels will increase (Nieto et al., 2000). Moreover, D-lactic acid, a metabolite of intestinal bacteria, is primarily found in the small intestine, but it will be released into the blood circulation when intestinal injury occurs (Li J. et al., 2021). Therefore, increased serum DAO and D-lactic acid levels can be considered as circulating markers for reflecting an injury degree of intestinal mucosal barrier. In the present study, serum D-lactic acid concentration and DAO activity were found to be decreased in weaned rabbits fed the CGA diet, indicating that CGA had beneficial effects on attenuating intestinal permeability of weaned rabbits. In addition, another meaningful discovery in the present study was that dietary CGA supplementation could also improve the concentration of TGF-α in the jejunum of weaned rabbits. TGF-α is another crucial molecule in maintaining the integrity of intestinal epithelial cells (Chen et al., 2019). This result further suggested the improved effect of CGA on maintaining the integrity of intestinal permeability in weaned rabbits.

Intestinal inflammatory is one of the activators causing intestinal barrier dysfunction (Wang et al., 2020). Luissint et al. (2016) reported that the inflammatory microenvironment could destroy the structure and function of epithelial cell-to-cell junctions, impairing the epithelial barrier and mucosal homeostasis. The inflammatory biomarkers, including TNF-α, IL-6 and IL-1β, were reported to be elevated in weaned animals (Hu et al., 2013; Ma et al., 2021). In the present study, we found that CGA decreased levels of IL-6 and IL-1β in serum and TNF-α in jejunum mucosa, and increased levels of IL-10 in serum and jejunum mucosa. Similar results were also found in previous research in weaned pigs (Chen et al., 2018b). Increased TNF-α concentration usually results in the dysfunction of intestinal mucosal barrier by amplifying local and systemic inflammation (Wang et al., 2019). Yu et al. (2021) pointed out that TNF-α and IL-6 could induce inflammatory activation and tight junction conformation change, and consequently increase the permeability of Caco-2 cell monolayer. Besides, IL-10, as one of the key anti-inflammatory cytokines, can inhibit immune responses to antigens and suppress the expression of pro-inflammatory factors such as TNF-α, IL-6, and IL-1β (Xu et al., 2020). A previous study in IL-10-deficient mice showed that IL-10-deficiency was a primary initiating event in chronic intestinal inflammation (Simon et al., 2016). Therefore, the results in current study indicated that CGA could alleviate the systemic and intestinal inflammatory responses of weaned rabbits by maintaining the cytokines at normal levels, further protecting the integrity of the intestinal barrier. Moreover, MHCII is one of the key molecules in the adaptive immune system, and plays an important role in presenting peptide antigens to T cell scrutiny (Reynisson et al., 2020). In the present study, CGA supplementation significantly increased the concentration of MHCII in the jejunum mucosa, further suggesting that CGA could improve the intestinal anti-inflammatory ability.

In addition, intestinal oxidative stress is closely related to the intestinal barrier dysfunction (Wang et al., 2020). Under normal physiological conditions, the oxidation and antioxidant defense systems are in dynamic equilibrium (Metwally et al., 2020). A previous study indicated that weaning could induce oxidative stress by disrupting the physiologic equilibrium of oxidants and antioxidants (Zhu et al., 2012). Therefore, antioxidant ability of intestinal tissues is important for animal growth. A previous study reported that MDA is an important index reflecting the degree of membrane lipid peroxidation (Cheng et al., 2020). In addition, CAT, one of the most important endogenous antioxidant enzymes, plays a key role in alleviating oxidative stress by converting hydrogen peroxide to H_2_O (David et al., 2008). In the present study, dietary CGA supplementation reduced the MDA contents, while decreased the activity of CAT in serum, indicating that CGA relieved the oxidative stress of weaned rabbits. In jejunal mucosa, the MDA contents was also reduced, but the content of T-AOC was dramatically increased when rabbits were fed CGA-supplemented diet. The value of T-AOC represents the ability of antioxidants to scavenge free radicals, which reflects the total antioxidant capacity of the body’s defense system and is closely related to the health of body (Tian et al., 2021). Han et al. (2010) pointed that CGA could inhibit cellular oxidative stress as a chemical protective antioxidant by enhancing the cellular antioxidant system’s ability. Therefore, according to those results, we speculated that CGA exhibited an alleviation effect on weaning stress-induced oxidative stress in weaned rabbits partly by improving the antioxidant capacity of the small intestine. But further studies are needed to obtain evidence of the antioxidant effect of CGA.

Based on previous results from Gonthier et al. (2003), dietary CGA is only absorbed at a rate of approximately 33% in the small intestine, and consequently, most dietary CGA is able to reach the large intestine. Gut health often refers to interaction between the intestinal wall barrier, the microbiota, and physiological and immunological components, which enable different animals to cope with internal and external stressors (Placha et al., 2022). The intestinal mucosa, serving as cellular barrier, is the primary location of interaction with external materials and bacteria (Perez-Lopez et al., 2016). An integrated and mature intestinal mucosa plays a vital role in suppressing the colonization and proliferation of pathogenic bacteria (Chen et al., 2021a). In the present study, we found that CGA supplementation increased alpha diversity indexes including observed species, Shannon index, Simpson index, Chao 1 index, and ACE index of cecal microbiota. Previous study in weaned piglet also demonstrated that dietary CGA supplementation increased Shannon index, Simpson index, Chao 1 index, and ACE index of cecal microbiota (Chen et al., 2019). Generally, the observed species was used to calculate unique OTUs, Shannon and Simpson indexes were used to measure community diversity, and Chao 1 and ACE indexes were used to estimate community richness (Li et al., 2020). The results in this study indicated that CGA supplementation could increase bacteria community diversity and richness in cecum of rabbits. The gut microbiota diversity is a reliable indicator of host health. Inflammatory response and oxidative stress were often accompanied by a decreased diversity of gut microbiota which was reproducibly observed in the individuals with gut microbiota dysbiosis (Vasquez et al., 2019). Moreover, we also found that rabbits in CGA group had higher abundances of *V9D2013_group*, *Monoglobus*, *Papillibacter*, *UCG-005*, and *Ruminococcus* than those in CON group. *V9D2013_group*, *Monoglobus*, *Papillibacter*, *UCG-005*, and *Ruminococcus* were butyrate-producing bacteria (Hao et al., 2022; Yan et al., 2022). Chen et al. (2019) has proven that CGA diet intake increases cecal concentrations of short-chain fatty acids (including acetate, propionate, and butyrate) in weaned piglets. Short-chain fatty acids, especially butyrate, produced by gut microbiota were reported to suppress inflammatory response and oxidative stress (Hu et al., 2013). Besides, *Monoglobus* was enriched in health individuals, and its abundance was positively correlated with CD4+ T cell counts (Li R. et al., 2022; Wang et al., 2022). CD4(+) T cells play crucial roles in adaptive immune response against pathogens (Yang et al., 2020). *Papillibacter* was considered as a kind of beneficial bacteria that enriched in healthy gut (Yasoob et al., 2021). Lower *Papillibacter* abundance was observed in the damaged cecum induced by heat stress in rabbits (Yasoob et al., 2021). *Ruminococcus* was one of the keystone bacteria of the rabbit cecal microbiota, and could degrade and convert complex polysaccharides into a variety of nutrients for the hosts (Bäuerl et al., 2014; La Reau and Suen, 2018). Therefore, CGA supplementation increased the abundance of beneficial bacteria in the cecum of rabbits. A previous study showed that the cecal microbiota of rabbit could influence the host growth and feed efficiency (Velasco-Galilea et al., 2021). Increased beneficial bacteria is conducive to shaping mucosal immunity, inhibiting the inflammatory response and oxidative stress, and maintaining mucosa integrity of the gut (Perez-Lopez et al., 2016; Li W. et al., 2021). Consistently, in the present study, the changes of bacterial abundances also showed significant correlations with the parameters related inflammatory response and oxidative stress in serum and small intestine of weaned rabbits. Above all, increased bacteria community diversity and richness and beneficial bacteria abundance might be another reason for the positive effect of CGA supplementation on intestinal epithelial barrier functions.

## Conclusion

In conclusion, dietary supplemented with 800 mg/kg CGA exerts growth-promoting effects by enhancing intestinal structural integrity, improving intestinal barrier and absorption function, as well as maintaining intestinal microbial homeostasis, which give insight into the protective effects of CGA on the growth retardant of weaned rabbits. These findings allow for a broader understanding of the growth-promoting effects of CGA on the weaned rabbits.

## Data availability statement

The datasets presented in this study can be found in online repositories. The names of the repository/repositories and accession number(s) can be found at: https://www.ncbi.nlm.nih.gov/, PRJNA848144.

## Ethics statement

The animal study was reviewed and approved by the Care and Use committee of Shandong Agricultural University (protocol code SDAUA-2021-050).

## Author contributions

JC, LL, and FL: conceptualization. JC, ZS, and RJ: data curation and project administration. JC and ZS: formal analysis. FL: funding acquisition. RJ and HZ: investigation. YL and HZ: methodology. ZS, RJ, and YL: resources. JC and ZS: software. LL and FL: supervision. JC and ZS: visualization. JC: writing—original draft. LL and FL: writing—review and editing. All authors contributed to the article and approved the submitted version.

## Funding

This work was supported by the Natural Science Foundation of Shandong Province (ZR2021QC108 and ZR2021MC043), Postdoctoral Science Foundation of Shandong Agricultural University (040/760077), the earmarked fund for CARS (CARS-43-B-1), Special Economic Animal Industry Technology System of Shandong Province (SDAIT-21-16) and Taishan Industry Leadership Project (TSCY20190107).

## Conflict of interest

YL and HZ were employed by Qingdao Kangda Food Co., Ltd.

The remaining authors declare that the research was conducted in the absence of any commercial or financial relationships that could be construed as a potential conflict of interest.

## Publisher’s note

All claims expressed in this article are solely those of the authors and do not necessarily represent those of their affiliated organizations, or those of the publisher, the editors and the reviewers. Any product that may be evaluated in this article, or claim that may be made by its manufacturer, is not guaranteed or endorsed by the publisher.
