# Supplementary material for: Chlorogenic acid improves growth performance of weaned rabbits via modulating the intestinal epithelium functions and intestinal microbiota
Source: Front Microbiol. 2022 Nov 7;13:1027101. doi: 10.3389/fmicb.2022.1027101 (PMC9676508; doi:10.3389/fmicb.2022.1027101)
Supplement: Supplementary file 1 [file Data_Sheet_1.docx]

**TABLE S1 |** Effects of chlorogenic acid supplementation in the diets of rabbits on the relative abundance of cecal microbiota in phylum level

| **Taxonomy, %** | **Treatments^1^** | | **SEM** | ***P* value** |
| --- | --- | --- | --- | --- |
|  | **CON** | **CGA** |  |  |
| Firmicutes | 34.91 | 40.28 | 3.01 | 0.391 |
| Bacteroidota | 29.45 | 27.26 | 1.84 | 0.570 |
| Verrucomicrobiota | 10.56 | 5.25 | 2.24 | 0.249 |
| Proteobacteria | 4.99 | 4.83 | 1.34 | 0.955 |
| unidentified_Bacteria | 7.56 | 11.11 | 1.18 | 0.139 |
| Actinobacteriota | 1.24 | 2.05 | 0.35 | 0.264 |
| Desulfobacterota | 2.52 | 1.67 | 0.40 | 0.307 |
| Euryarchaeota | 0.61 | 0.26 | 0.12 | 0.161 |
| Spirochaetota | 0.05 | 0.09 | 0.02 | 0.397 |
| Synergistota | 0.32 | 0.28 | 0.10 | 0.829 |

Values are mean and SEM (*n* = 8). Differences were considered statistically significant when *P* < 0.05.

^1^ CON, rabbits fed a basal diet; CGA, rabbits fed a basal diet supplemented with 800 mg/kg chlorogenic acid.

**TABLE S2 |** Effects of chlorogenic acid supplementation in the diets of rabbits on the relative abundance of cecal microbiota in genus level

| **Taxonomy, %** | **Treatments** | | **SEM** | ***P* value** |
| --- | --- | --- | --- | --- |
|  | **CON** | **CGA** |  |  |
| *Akkermansia* | 10.55 | 5.23 | 2.24 | 0.248 |
| *NK4A214_group* | 6.73 | 5.65 | 1.24 | 0.679 |
| *Escherichia-Shigella* | 1.86 | 2.96 | 1.16 | 0.653 |
| *Christensenellaceae_R-7_group* | 6.14 | 3.53 | 1.18 | 0.284 |
| *Desulfovibrio* | 2.34 | 1.46 | 0.41 | 0.292 |
| *Ruminococcus* | 1.52 | 2.61 | 0.33 | 0.095 |
| *Bacteroides* | 2.05 | 1.34 | 0.35 | 0.326 |
| *Prevotella* | 0.39 | 1.08 | 0.29 | 0.242 |
| *dgA-11_gut_group* | 0.87 | 0.65 | 0.27 | 0.692 |
| *V9D2013_group* | 0.86 | 1.71 | 0.18 | 0.012 |
| *Alistipes* | 1.77 | 1.44 | 0.21 | 0.456 |
| *Ralstonia* | 1.00 | 0.35 | 0.24 | 0.173 |
| *Bombella* | 1.14 | 0.68 | 0.19 | 0.242 |
| *Lachnoclostridium* | 0.68 | 0.16 | 0.19 | 0.179 |
| *[Eubacterium]_siraeum_group* | 0.99 | 1.19 | 0.11 | 0.410 |
| *Pseudomonas* | 0.84 | 0.58 | 0.17 | 0.478 |
| *Methanobrevibacter* | 0.51 | 0.21 | 0.11 | 0.179 |
| *Synergistes* | 0.29 | 0.22 | 0.09 | 0.731 |
| *Rikenellaceae_RC9_gut_group* | 0.27 | 0.54 | 0.14 | 0.327 |
| *unidentified_Muribaculaceae* | 0.03 | 0.01 | 0.01 | 0.501 |
| *Monoglobus* | 0.60 | 0.88 | 0.06 | 0.023 |
| *Rubrobacter* | 0.00 | 0.16 | 0.07 | 0.225 |
| *Lachnospiraceae_NK4A136_group* | 0.79 | 0.73 | 0.05 | 0.550 |
| *Papillibacter* | 0.23 | 0.40 | 0.04 | 0.010 |
| *Blautia* | 0.51 | 0.31 | 0.06 | 0.110 |
| *Turicibacter* | 0.14 | 0.02 | 0.06 | 0.371 |
| *Phascolarctobacterium* | 0.21 | 0.30 | 0.07 | 0.537 |
| *Marvinbryantia* | 0.36 | 0.18 | 0.06 | 0.149 |
| *UCG-005* | 0.31 | 0.52 | 0.04 | 0.009 |
| *Eisenbergiella* | 0.18 | 0.04 | 0.05 | 0.202 |

Values are mean and SEM (*n* = 8). Differences were considered statistically significant when *P* < 0.05.

^1^ CON, rabbits fed a basal diet; CGA, rabbits fed a basal diet supplemented with 800 mg/kg chlorogenic acid.


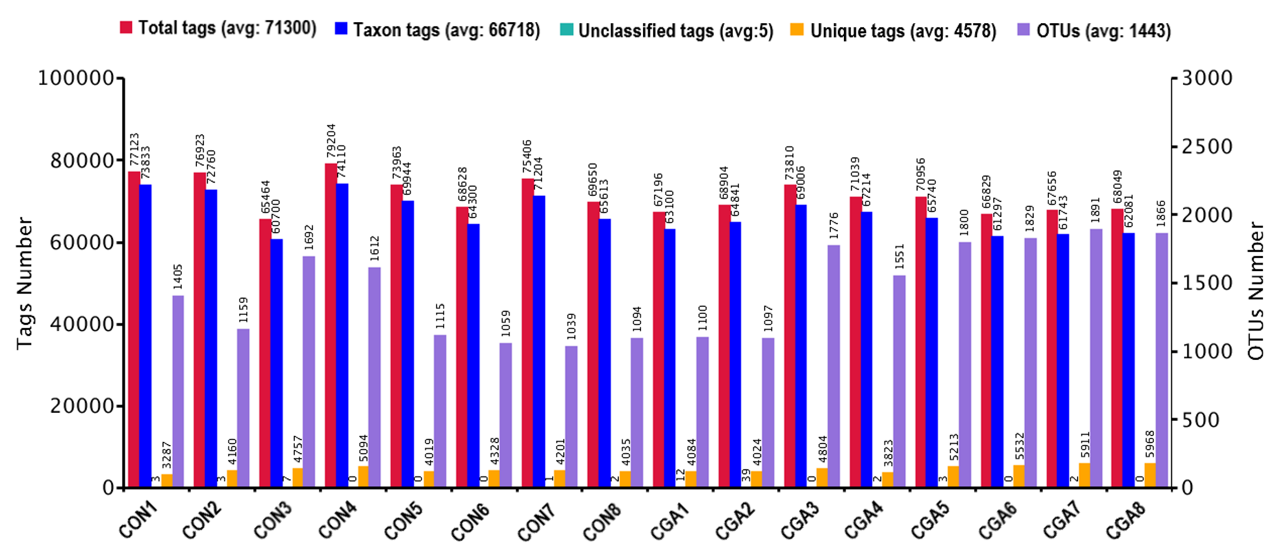


**Figure 1.** Operational taxonomic unit (OTU) clustering and annotation per sample. CON 1, 2, 3, 4, 5, 6, 7, 8 are cecal digesta samples from rabbits fed with a basal diet; CGA 1, 2, 3, 4, 5, 6, 7, 8 are cecal digesta samples from rabbits fed with a basal diet supplemented with 800 mg/kg chlorogenic acid.
